# Treatment demand for cannabis use problems: analyses of routine data from 30 European countries

**DOI:** 10.1007/s00406-024-01840-w

**Published:** 2024-06-12

**Authors:** Jakob Manthey

**Affiliations:** 1 Centre of Interdisciplinary Addiction Research (ZIS), Department of Psychiatry and Psychotherapy, University Medical Center Hamburg-Eppendorf (UKE), Martinistraße 52, 20246 Hamburg, Germany; 2 Department of Psychiatry, Medical Faculty, University of Leipzig, Semmelweisstraße 10, 04103 Leipzig, Germany

**Keywords:** Cannabis, Treatment demand, Treatment need, Treatment gap, Europe, Legalisation

## Abstract

Cannabis use and treatment demand has risen in the past decade. Previous analyses of treatment demand are limited by methodological constraints or are outdated. Cross-country differences and trends in cannabis treatment demand are described using data from the European Monitoring Centre for Drugs and Drug Addiction. Two novel indicators are employed: firstly, the cannabis-attributable treatment fraction (CATF) is obtained by dividing the number of treatment entrants for cannabis use problems by the number of treatment entrants for any substance use problem, accounting for possible changes in the reporting system. Secondly, comparing the number of treatment entrants for cannabis use problems to the number of people who use cannabis (near) daily yields the treated-user-ratio (TUR), which considers a proxy for treatment need (frequent use). Across 30 countries with available data, the importance of cannabis in European treatment facilities varies greatly (CATF: min = 3%; max = 65%), with lower estimates in Eastern European countries. Across 20 countries with complete data, the CATF has risen from 29.4% in 2013 to 37.1% in 2020. The TUR calculated on 26 countries suggests that about 3 in 100 frequent users have sought treatment for their cannabis use problems. Over time, treatment demand has increased at a slower pace than treatment need in most countries. One in three treatment entrants for substance use problems in Europe are due to cannabis, with large variations between countries. There are indications for a widening treatment gap for cannabis use problems. In countries liberalising cannabis laws, monitoring changes in treatment access and demand is warranted.

**Supplementary Information:**

The online version contains supplementary material available at 10.1007/s00406-024-01840-w.

## Introduction

In various European countries, increasingly liberal approaches to cannabis regulation can be observed. According to a 2017 report, cannabis possession was decriminalised in Luxembourg, Croatia, Portugal, and Slovenia [8]. Other countries with de facto decriminalisation include the Netherlands and Spain. In Malta and Luxembourg, home cultivation has been allowed recently and Germany may follow in 2024 [7, 33].

Parallel to these political reforms, an upward trend regarding cannabis consumption and treatment demand for cannabis-related problems can be observed [13, 19, 20]. According to a report on ‘Treatment of Cannabis-related Disorders in Europe’ published by the European Monitoring Centre for Drugs and Drug Addiction (EMCDDA; [13]), the number of cannabis-related treatment entrants in Europe increased between 2006 and 2012, whereas the number of treatment admissions for cocaine and heroin (the two other major drug classes concerning treatment) decreased. Consequently, cannabis makes up the primary drug of concern for about 30% of all first-time treatment entrants in eight European Union countries, including the most populous countries Germany, France and Poland [13]. More recent data from Germany suggest that this trend has continued for hospital admissions [15] but also for diagnoses in outpatient settings [21].

These increasing trends appear to contrast observations in North America, where the regulations for recreational cannabis are increasingly loosened. In the US, the first legal commercial market was set up in Colorado in 2013 and followed by more than a dozen states in the following decade. Here, the absolute number of admissions to treatment facilities for cannabis as main drug increased before 2012 but declined thereafter. This pattern was confirmed when examining the share of all substance use admissions that were attributable to cannabis [29]. Overall, it seems that treatment admissions due to cannabis use problems decrease while cannabis use prevalence increases [24]. Moreover, the declining treatment demand for chronic cannabis use problems was more pronounced in legalising US states, even after controlling for reduced police and court referrals post-legalisation [23].

Overall, it appears that European countries see an increase in treatment demand while US states experience the opposite. However, there are several knowledge gaps. First, the latest comprehensive analysis of European treatment data only extends up to 2012 [13] or 2014 [25]. Second, analysing only the absolute number of treatment entries may be biased because the reporting system or coverage of facilities changes over time. Third, to fully understand treatment demand, the data need to be contextualised with treatment *need*, i.e., the number of persons requiring treatment for use problems. There is no standard way to define the need for cannabis treatment but *objective* treatment need is usually defined by meeting the diagnostic threshold for a cannabis use disorder (see e.g., [36]), however, this may differ from *subjective or perceived* treatment need [3, 36].

In this study, I aim to close those knowledge gaps by updating previous analyses of the European treatment demand data [13, 25] and examining recent trends of treatment demand for cannabis problems across the continent and between countries with two novel indicators.

## Methods

### Data sources

As part of the ‘treatment demand indicator’ (TDI), the EMCDDA compiles country-specific information on drug treatment demand [9]. Importantly, there are recognised differences “in national monitoring systems, coverage and definitions”, (see https://www.emcdda.europa.eu/data/stats2022/methods/tdi), which should be acknowledged when comparing countries.

At its core, the TDI contains annual counts of treatment entrants (i.e., individuals seeking drug treatment) for various types of illegal drugs by year, sex, and drug type (cannabis vs. any drug). In Supplementary Fig. 1, the changes in the number of treatment entrants for cannabis and any drugs are illustrated.

Moreover, the TDI gives additional drug treatment information, e.g., the type of referral by drug type. Across all countries, the predominant type of treatment referral is self-referral but legal referrals are also prevalent in several countries. Supplementary Fig. 2 shows country variation in referral types and the time trend in legal referrals is highlighted in Supplementary Fig. 3. Information on the type or severity of cannabis use problems or the type of treatment provided are not available.

All data presented in this study refer to the population aged 15–64 living in the 27 European Union countries and, in addition, Norway, Turkey and the United Kingdom (total number of countries: n = 30). For each country, the most recent data are reported in Table 1.

**Table 1:** Key data from European countries based on the last available year

Country	Year	Treatment entrants (n)		CATF (%)	Treatment need^1^ (%)	Treatment need^1^ (n)	TUR
		Any drug	Cannabis				
Austria	2020	4531	1198	26.4	0.8	47,352	2.5
Belgium	2018	11,887	3808	32.0	1.3	96,417	3.9
Bulgaria	2021	1915	122	6.4			
Croatia	2019	6858	1039	15.2	1.3	35,380	2.9
Cyprus	2019	982	490	49.9	0.4	3404	14.4
Czechia	2020	7283	1072	14.7	0.2	13,453	8.0
Denmark	2021	7383	2917	39.5	0.9	33,492	8.7
Estonia	2021	485	27	5.6			
Finland	2018	676	124	18.3	0.7	23,887	0.5
France	2017	58,077	28,205	48.6	1.9	768,977	3.7
Germany	2021	44,404	25,852	58.2	1.5	812,127	3.2
Greece	2015	4087	789	19.3	0.3	19,910	4.0
Hungary	2019	4579	2942	64.2	0.2	11,259	26.1
Ireland	2021	10,408	2228	21.4			
Italy	2017	46,586	10,155	21.8	1.1	406,072	2.5
Latvia	2015	751	175	23.3	0.2	2463	7.1
Lithuania	2021	465	35	7.5	0.1	1790	2.0
Luxembourg	2019	381	128	33.6	1.2	5130	2.5
Malta	2021	1990	291	14.6			
Netherlands	2015	10,987	5202	47.3	1.5	163,003	3.2
Norway	2021	5839	1622	27.8	0.4	14,034	11.6
Poland	2014	7186	2483	34.6	0.2	49,515	5.0
Portugal	2016	3294	1066	32.4	3.0	200,445	0.5
Romania	2019	4283	2336	54.5	0.3	39,103	6.0
Slovakia	2019	3295	699	21.2	0.2	7776	9.0
Slovenia	2018	219	15	6.8	0.7	9677	0.2
Spain	2017	46,799	12,932	27.6	3.7	1,142,407	1.1
Sweden	2021	32,826	3263	9.9			
Türkiye	2021	9353	1619	17.3			
United Kingdom	2018	114,752	25,103	21.9	0.9	398,222	6.3

Empty cells indicate no data available

*CATF* cannabis-attributable treatment fraction, *TUR* treated-user-ratio

^1^Treatment need is defined as the prevalence (%) and absolute number (n) of (near) daily cannabis users

### Definition of treatment demand indicators

Treatment demand for cannabis problems may be operationalised by the absolute number of treatment entrants. Cross-country differences in treatment systems and changes in documentation manuals over time could severely limit the interpretation of absolute numbers. To minimise possible bias in the interpretation of data and to facilitate diverse perspectives on treatment demand, two new indicators for monitoring treatment demand were employed:

1. The Cannabis-Attributable Treatment Fraction (CATF) contextualises the treatment demand for cannabis with treatment demand for other drugs. This fraction implicitly controls for cross-country differences and within-country changes in the reporting system that can affect the documented number of treatment entrants for cannabis and other drugs.
2. The treated-user-ratio (TUR) contextualises treatment demand for cannabis with the need for cannabis treatment. This ratio implicitly controls for cross-country differences and within-country changes in the levels of cannabis use.

### Cannabis-attributable treatment fraction (CATF)

To describe changes in treatment demand, possible biases in the reporting system should be accounted for. As the number of treatment facilities can change over time (for European data, see Appendix of [20]), an increase in the number of treatment entrants may merely reflect an increase in the number of treatment units reported to the EMCDDA and not an actual increase in the treatment demand.

Analogous to a previous analysis of cocaine treatment demand in ten European countries [2], the number of cannabis-related treatment entrants was divided by the number of all drug treatment entrants. The resulting indicator is the Cannabis-Attributable Treatment Fraction (CATF), which measures the contribution that cannabis makes to the overall treatment demand in each country at a given time. By contrast to the cannabis treatment rate (i.e., number of persons receiving cannabis treatment per 100,000 population), the CATF accounts for major changes in the TDI within countries over time. For instance, a change in the number of treatment units contributing data to the TDI over time (e.g., because a major clinic closed or stopped reporting data to the TDI) can have a considerable impact on the treatment rate, but a smaller impact on the relative contribution that cannabis makes to all drug treatments. This makes the CATF a more robust indicator for monitoring purposes.

### Treated-user-ratio (TUR)

A key and perhaps most obvious determinant of the demand for cannabis-related treatment is the number of people in need of such treatment. To define treatment need, one would preferably refer to cannabis use disorder—the clinical diagnosis given to people using cannabis who experience loss of control, craving as well as social or health consequences from continued use of cannabis.

Because information on cannabis use disorder prevalence is not available, I rely on the prevalence of frequent use, i.e., daily or near-daily use, as a proxy for cannabis use disorder. Frequent use is a key risk factor for developing cannabis-related problems such as cannabis use disorder [30] or psychotic symptoms [31]. Based on longitudinal studies, the risk for cannabis dependence among weekly or daily users was estimated at 33%, compared to 9% for less frequent use [17]. In a US-based sample, the average number of use days in the past month was 6.5 for people without and 19.8 for people with a cannabis use disorder [14]. It should be acknowledged that frequent use is not a diagnosis or an objective measurement of treatment need but can only be a proxy that should be interpreted with some caution.

In a 2015 EMCDDA report, frequency of use was also used as a proxy for treatment need [13]. Here, a treated-user-ratio (TUR) was calculated by dividing the number of cannabis-related treatment entrants by the estimated number of (near) daily cannabis users and multiplied by 100 to produce the number treated per 100 (near) daily cannabis users. The TUR compares treatment demand with the number of frequent cannabis users—as proxy for treatment need.

It should be acknowledged that the TUR relies on estimates of use frequency from general population surveys, which are not conducted in all countries regularly. Accordingly, the TUR was only available for select years and not for all countries. Data on population size for each country were obtained from the UN Population Prospects, 2022 revision [26], and were applied to obtain the number of (near) daily users.

All data processed in this study were downloaded from publicly available sources and processed in R [27]. The data and R codes are publicly available (https://figshare.com/s/9c2836155a80c0c2eddf).

## Results

### Cross-country differences in treatment demand for cannabis use problems

**Fig. 1:**
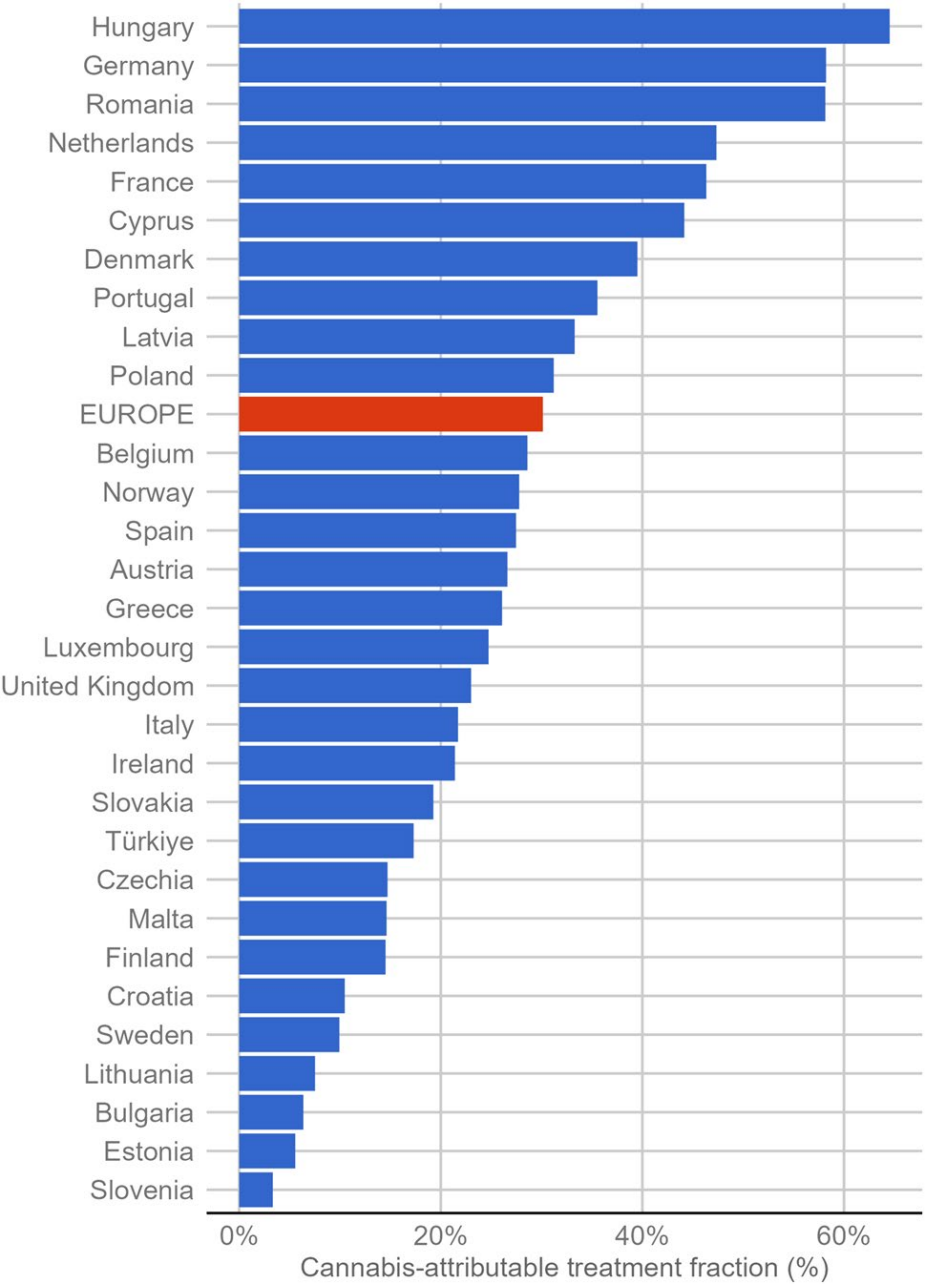
Cannabis-attributable treatment fraction (CATF) in European countries and averaged (red bar) based on last data point available (2021 in all countries except for Czechia (2020), France (2020), Spain (2020), the Netherlands (2015), and United Kingdom (2019))

Based on the most recent data available from all n = 30 countries (primarily from 2021), the share of cases with cannabis-related problems as main drug, expressed as CATF, varied considerably across countries (see Fig. 1). Cannabis treatments comprised up to 10% of all cases in Slovenia (3%), Estonia (6%), Bulgaria (6%), Lithuania (8%) and Sweden (10%). Except for Sweden, the number of absolute treatment entries for cannabis in these countries ranged between 7 and 122 (Sweden: 3263). In Romania (58%), Germany (58%), and Hungary (65%), more than every second drug treatment entrant sought help for cannabis-related problems. The most recent CATFs for each country are reported in Table 1.

### European trends in treatment demand for cannabis use problems

Analyses of the CATF trends were restricted to a subset of n = 20 countries that provided complete data between 2013 and 2020 (for data availability by country, see Supplementary Fig. 1). These 20 countries comprised the most populous, accounting for 90% of all European cannabis treatment entrants in 2020, therefore providing robust data. Between 2013 and 2020, the number of treatment entrants for any drug problems first increased from about 310,000 in 2013 to 350,000 in 2015, before subsequently decreasing and reaching a minimum of around 250,000 in 2020 (see also Table 2 for gender-stratified numbers). The absolute number of cannabis-specific treatment entrants followed a similar trend. However, contrasting cannabis- to any drug-related treatment entrants shows that the CATF has slightly increased, rising from 29.4% in 2013 to 37.1% in 2020. This trend was similar for both women (22.5–32.3%) and men (31.1–38.2%).

**Table 2:** Number of any drug-related and only cannabis-related treatment entrants in 20 European countries between 2013 and 2020

Women	Any drug-related treatment	Cannabis-related treatment	CATF (%)
2013	58,418	13,146	22.5
2014	58,148	14,872	25.6
2015	59,624	15,868	26.6
2016	56,842	15,365	27.0
2017	48,136	13,831	28.7
2018	44,721	14,295	32.0
2019	45,432	14,978	33.0
2020	41,416	13,361	32.3
Men			
2013	250,728	77,952	31.1
2014	251,915	85,578	34.0
2015	256,263	90,982	35.5
2016	249,742	89,661	35.9
2017	219,530	80,305	36.6
2018	202,610	80,625	39.8
2019	203,790	80,878	39.7
2020	178,591	68,158	38.2

Data shown is based on the following 20 countries: Austria, Belgium, Cyprus, Finland, France, Germany, Greece, Hungary, Ireland, Italy, Lithuania, Luxembourg, Malta, Norway, Poland, Portugal, Romania, Slovakia, Slovenia, Spain, United Kingdom

*CATF* cannabis-attributable treatment fraction

### Treatment demand vs. treatment need

**Fig. 2:**
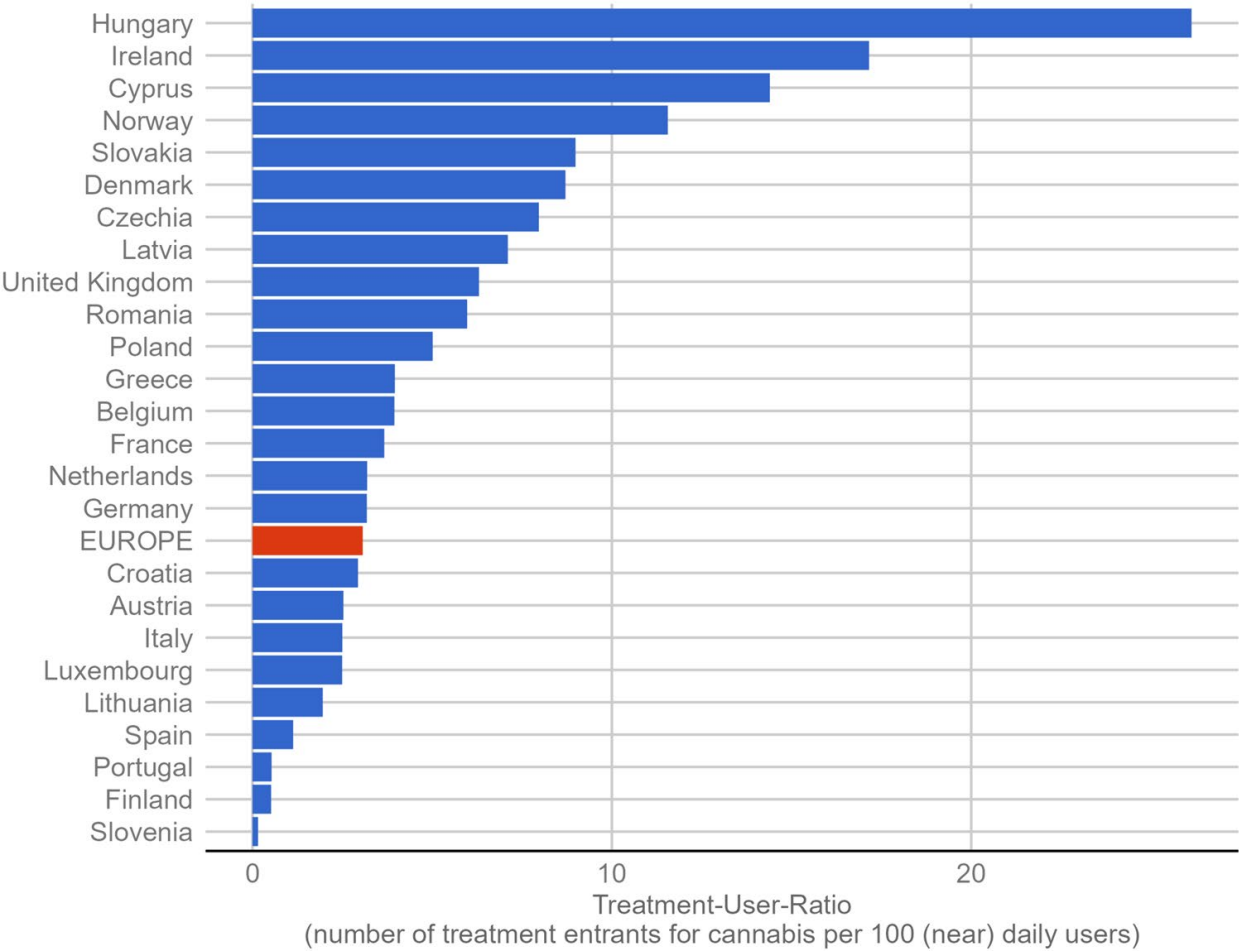
Treatment-User-Ratio (TUR) in European countries and averaged (red bar) based on the last data point available (between 2011 and 2021, median: 2018)

In estimating the unmet treatment need, the ratio of treated to (near) daily users—expressed as treatment-user-ratio (TUR)—was analysed. For this indicator, country- and year-specific information on (a) the number of treatment entrants with cannabis as main drug was matched with (b) the number of (near) daily cannabis use. Based on the most recent data available from n = 26 countries, 264,946 cannabis treatment entrants were registered. This compares to an average prevalence of (near) daily cannabis use of 1.3% or 4,317,451 persons. Across Europe, the TUR was 3.1, meaning that for 100 (near) daily users 3.1 treatment entrants were registered. As illustrated in Fig. 2, the TUR varied largely across countries, ranging from 0.2 in Slovenia to 26 in Hungary. The TURs for each country are reported in Table 1*.*

**Fig. 3:**
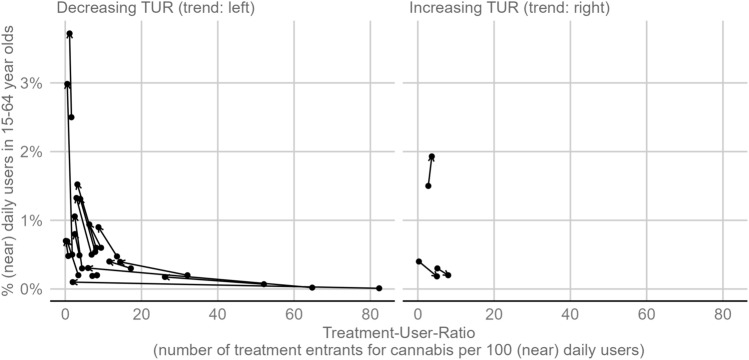
Treatment-User-Ratio (TUR; x-axis) plotted against the prevalence of (near) daily use (y-axis). Each line represents one of n = 18 countries and each dot indicates one year (arrows give the time order). Countries showing TUR decreases are plotted in the left half (Austria, Belgium, Croatia, Cyprus, Denmark, Finland, Germany, Hungary, Italy, Latvia, Lithuania, Norway, Portugal, Romania, Slovenia, Spain, United Kingdom) and countries showing TUR increases are plotted in the right half of the figure (Czechia, France, Poland)

For a trend analysis, the data were limited to countries with at least two years of information available to calculate the TUR. A total of 20 countries were included, spanning from the years 2010 to 2016 (median: 2012), and from 2014 to 2021 (median: 2018). As illustrated by left-leaning arrows in the left half of Fig. 3, a decreasing TUR was observed in 15 out of 18 countries. In those countries, there have been fewer treatment entrants recorded per 100 (near) daily users in the observed years. In contrast, only three out of 20 countries were observed to show an increasing number of treatment entrants recorded per 100 (near) daily users, as illustrated by right-leaning arrows in the right half of Fig. 3.

In addition to the change in the TUR plotted on the x-axis, Fig. 3 also shows changes in the prevalence of (near) daily users. As indicated by the arrows pointing upwards, the prevalence of (near) daily cannabis use has increased in most countries. This suggests that the number of (near) daily users is growing at a faster rate than the number of treatment entrants.

## Discussion

The present analyses highlight that the share of substance use treatment entrants who seek help for cannabis use problems has increased slightly between 2013 and 2020 on average in European countries. It is estimated that cannabis problems are the main reason to seek help for about one-third of treatment entrants. While the rise in cannabis-related treatment entrants is larger than for other drugs, the treatment demand increases at a slower pace than the prevalence of (near) daily cannabis use. If the number of (near) daily users is a reasonable proxy for changes in treatment needs over time, then the treatment gap is growing in most European countries.

### Limitations

Analyses of routine data can only be as accurate as the documentation and reporting system. The restricted temporal data availability for some countries (e.g., no data from the UK after 2019) has constrained the analyses to subsets of countries, limiting the generalisability of the presented findings. Analysing treatment demand for cannabis use problems requires a common definition of treatment and cannabis use problems to be applied across all countries, but it is unclear to what degree existing guidelines [12] are put into practice. Moreover, the treatment systems largely differ between countries, with some mostly reporting data from outpatient facilities and other referring mostly to interventions delivered in prisons, low-threshold treatment facilities or hospitals [11]. Based on the data available, it cannot be ascertained what kind of services are effectively delivered in which settings to people seeking help for their cannabis use problems. Similarly, further research is required to assess to which degree the increasing trend in the CATF is due to declining use of other drugs (for changes in any drug treatment trends, see Supplementary Fig. 1) or changes in treatment availability.

In absence of more accurate information, treatment need was measured by survey data on the number of people who used cannabis (near) daily. However, (near) daily cannabis use is not a clinical diagnosis but indicates a potential risk for developing a cannabis use disorder [5, 30]. These prevalence data were derived from (general population) surveys which may be distorted by sampling bias (e.g., under-sampling people who use drugs, not surveying persons with no fixed address), response bias and stigma (e.g., not acknowledging frequent cannabis use because of its illegal status). One recent study showed that common survey techniques largely underestimate (frequent) cannabis use in Sweden [1]. Lastly, the frequent use of cannabis may lead to problems later in life, but this potential time lag is not considered in these analyses.

### Implications

The fraction of cannabis to any-drug treatment demand (expressed as CATF) varies considerably across the European continent. Generally, the CATF is rather low in many Eastern European countries, suggesting that other substances are responsible for a greater share of treatment demand in this region. Conversely, cannabis makes up a sizeable share of entrants in Central and Western European countries. This pattern generally compares to the prevalence of use, which is highest in France, Spain, and neighbouring countries, while cannabis use remains rather uncommon in Eastern European countries [20].

While more information on those entering treatment and the characteristics of treatment systems will be required to fully understand the identified country differences, the two indicators CATF and TUR together provide important insights. For instance, the CATF in Portugal is slightly above the European average, with about 1 out of 3 treatment entrants receiving support for cannabis problems. However, only about 1 in 20 people who use cannabis (nearly) daily are estimated to have received treatment—lower than in most other countries. This gap could be explained in various ways. First, a sizeable number of people in treatment may not be covered in the TDI due to the limited coverage of the TDI documentation system in Portugal. While current information on treatment demand coverage in Portugal is not available, the share of cannabis treatment entrants referred by legal sources is relatively high in this country (Portugal: between 32 and 53%; European average: between 19 and 27%; see Supplementary Figs. 2 and 3). It should be noted that cannabis possession is decriminalized in Portugal but violations against drug laws can still be sanctioned with coerced treatment. Considering the liberal cannabis policies, the high share of legal referrals could indicate that not all treatment settings, especially medical settings with high rates of self-referrals, are appropriately covered in the TDI data from Portugal. Second, the prevalence of (near) daily cannabis use is higher in Portugal than in most other European countries. A higher prevalence of (near) daily cannabis use could first indicate a higher treatment need in this country. Alternatively, it is also conceivable that the comparably liberal drug policy environment in Portugal has normalized cannabis use, which may have minimized reporting biases in surveys. Assuming that users in Portugal are more willing to disclose sensitive behaviour than users in countries with more repressive environments, this would introduce a bias in the treatment need estimator as employed in this study.

This example highlights the need to view treatment demand from different perspectives. With both the CATF and the TUR, key (but not all) determinants of treatment demand are controlled so each indicator provides a different perspective on the same problem. The limitation of each indicator is that their interpretation depends on secondary data, i.e., consumption and treatment for other drugs (CATF; see also Supplementary Fig. 1) and cannabis treatment need (TUR). However, the combination of both indicators provides for a more complete picture of treatment demand for cannabis problems.

To gain a comprehensive understanding of the differences in the CATF and TUR among countries, it is necessary to gather more detailed information regarding the population who are registered as treatment entrants. Specifically, it would be of interest to ascertain the number of individuals who fulfil the diagnostic requirements for a cannabis use disorder and the settings (e.g., hospitals, general practitioners) where treatment is provided. Additionally, it is crucial to comprehend the range of services available to individuals and the duration of treatment. Lastly, policy approaches will need to be considered, as discussed for Portugal above. The large variations in legal drug referrals across countries (see Supplementary Fig. 2) as well as the increasing trend over time (Supplementary Fig. 3) may explain country-specific trends, which should be followed up in future research.

There are several hypotheses for the growing significance of cannabis in substance treatment facilities in Europe. First, the treatment demand follows the trends in treatment need, approximated using the (near) daily use prevalence. Importantly, the rising treatment demand does not seem to keep up with the rising treatment need, resulting in a possibly widening treatment gap. Second, changes in treatment referrals could contribute to an apparent increase in treatment demand. According to the latest available EMCDDA data, 26% of cannabis treatment entrants were legal referrals, i.e., from police, probation, or courts (see also Supplementary Fig. 2). Across all substances, only 15% were legal referrals [10]. The rising importance of legal referrals for cannabis (see Supplementary Fig. 3) may be one of the drivers for increased cannabis treatment demand observed in many countries. Third, the rising treatment demand could be explained in part by the increased use of synthetic cannabinoids. Overall, only a small minority of people report using synthetic cannabinoids on purpose [18], however, the risks for users are likely greater when exposed to this heterogeneous class of substances [4]. Robust data on treatment entrants for synthetic cannabinoids is not available, disallowing testing this hypothesis. Based on the available data from 2021, there were 685 treatment entrants for synthetic cannabinoids recorded in 15 countries, contrasting to 14,777 any-cannabis entrants (4.6%) [10]. The share of synthetic among any cannabis treatment entrants exceeds 5% in Bulgaria, Malta, and Turkey.

I can only speculate on reasons why CATF is increasing while TUR is decreasing. In US-based household surveys, a declining trend in the prevalence of cannabis use disorder among (near) daily cannabis users was observed between 2002 and 2016 [32], suggesting declining treatment demand among frequent users. It has been hypothesized that policy changes, risk perception, and reporting biases could explain these findings and it cannot be excluded that similar trends are occurring in European countries. I want to add additional thoughts on the discrepancy of trends in CATF and TUR. First, the prevalence of (near) daily cannabis use may increasingly capture groups that are considerably less likely to require treatment for cannabis use problems. This includes people using cannabis (primarily) for medical purposes and those using (primarily) low THC/high CBD products. Following legislative changes in several countries (e.g., Czechia 2013; Germany 2017), medical cannabis use may rise considerably in European countries but empirical data is scarce. General population surveys I am aware of do not explicitly distinguish medical from recreational use (e.g., Germany: [28]; Austria: [35]; Spain: [34]). As for low-THC/high-CBD products, the rising popularity has resulted in about 1 in 10 adults in Germany reporting using those products, with a sizeable share (18%) reporting daily consumption [16]. In Austria, the prevalence of high-THC and low-THC/high-CBD products was comparable in 2020 (3–5%: [35]). If population surveys do not explicitly remove those users from prevalence estimates, then the increasing prevalence of (near) daily use may be driven by increased medical use or use of low-THC/high-CBD products, diluting the robustness of the (near) daily use prevalence as an indicator for treatment need. A last reason why the TUR is decreasing during times of increasing CATF may be related to changes in documentation of treatment demand. As shown in Supplementary Fig. 1, there has been a substantial drop in the number of documented treatment entrants for cannabis and other drugs in Germany after 2015, driven by the introduction of a new documentation manual (“Kerndatensatz 3.0”, see [6]). With nearly 26,000 treatment entrants in 2021, Germany makes up a sizeable share of all treatment entrants documented across Europe, so, likely, the drop in the number of treatment entrants observed in 2017 across all countries (see Table 2) is driven by the documentation change in Germany. With the prevalence of near-daily use rising and the absolute number of treatment entrants falling, this results in a declining TUR for Germany. Considering these methodological problems, it is important to have robust and comparable estimates of treatment need, i.e., the prevalence of cannabis use disorders, in European countries.

Overall, my findings suggest that European countries also observe a declining trend of absolute treatment entrants for cannabis and other drugs as observed in the US [24]. However, in Europe, the relative importance of cannabis in substance use treatment settings continues to rise, while it decreases in the US, especially in regions where cannabis has been legalised [23]. It appears likely that the policy environment impacts treatment demand, directly or indirectly. While an increasing number of European countries are liberalising their cannabis regulations, we are only beginning to understand how the policy environment impacts consumption, risk perception, and treatment for cannabis problems [22].

## Conclusion

The available data point to the increasing importance of cannabis in European substance use treatment settings. Possibly, the growth of treatment need exceeds that of treatment demand, resulting in a widening treatment gap. Liberalising the cannabis policy environments in European countries may impact those trends and warrants careful monitoring.

## Supplementary Information

Below is the link to the electronic supplementary material.

Supplementary file1 (DOCX 677 KB)

## Data Availability

The data and R codes are publicly available (https://figshare.com/s/9c2836155a80c0c2eddf).
