# Supplementary material for: Treatment demand for cannabis use problems: analyses of routine data from 30 European countries
Source: Eur Arch Psychiatry Clin Neurosci. 2024 Jun 12;275(2):355–63. doi: 10.1007/s00406-024-01840-w (PMC11910416; doi:10.1007/s00406-024-01840-w)
Supplement: Supplementary file 1 — Supplementary file1 (DOCX 677 KB) [file 406_2024_1840_MOESM1_ESM.docx]

**Supplementary Information**

**Title:** Treatment demand for cannabis use problems: analyses of routine data from 30 European countries

**Author:** Jakob Manthey


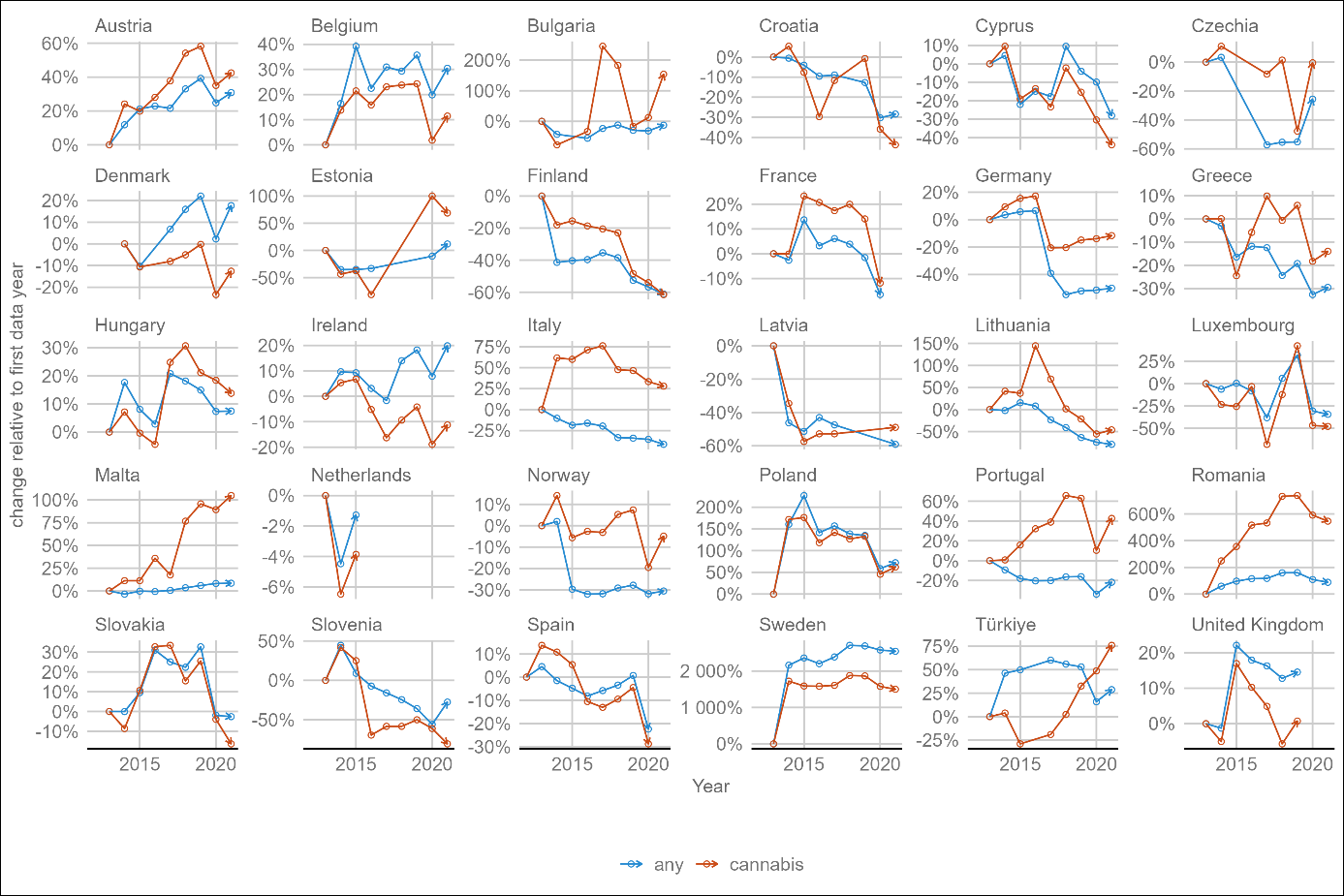


**Supplementary Figure 1.** Relative change in the number of treatment entrants for any drug (blue) and cannabis (red) problems in 30 European countries. When the red line is above the blue line, this indicates a shift towards cannabis cases, i.e., an increase in the cannabis attributable treatment fraction. Each circle indicates one year of data availability.


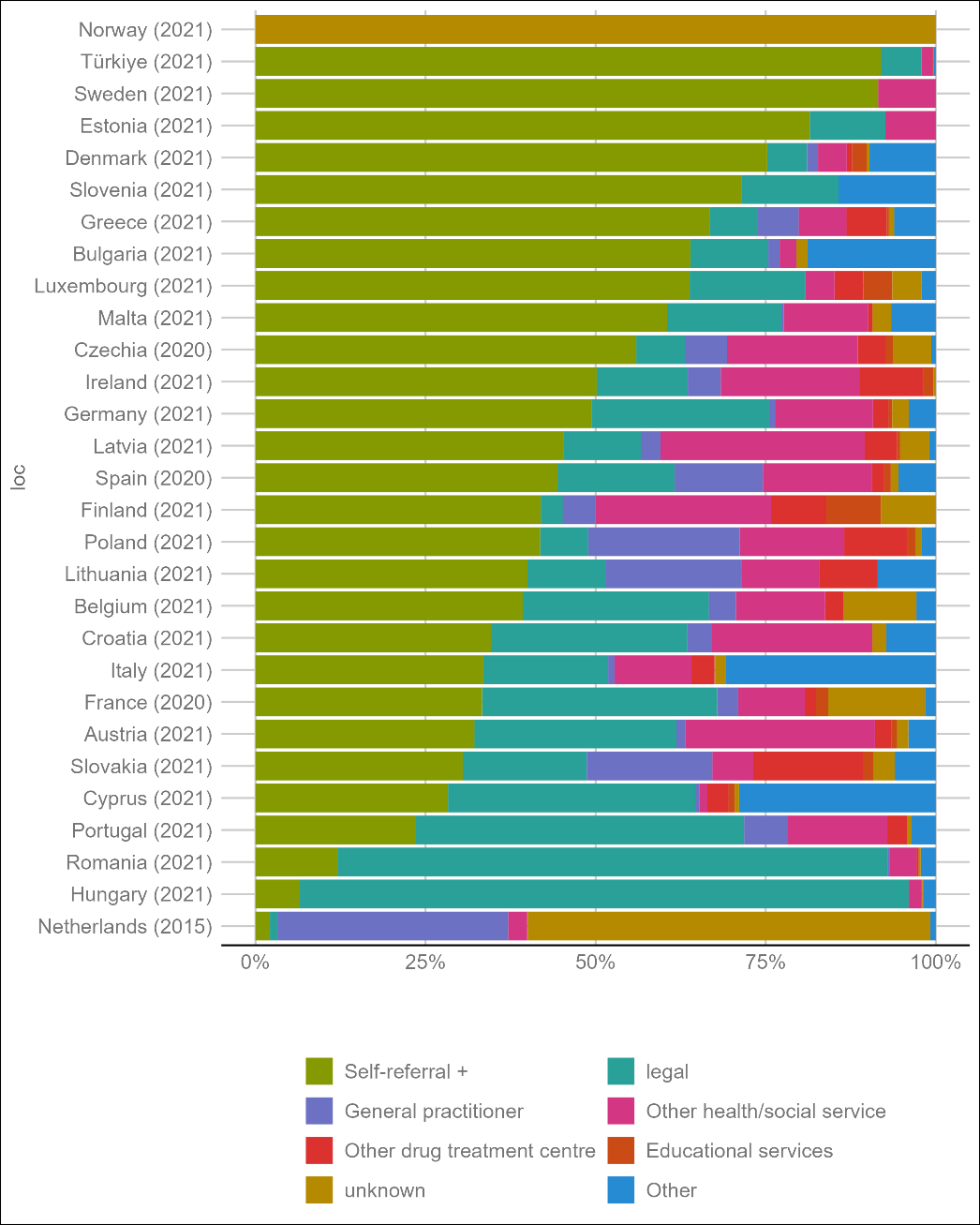


**Supplementary Figure 2** – The share of treatment entrants for cannabis problems by type of referral. Self-referral +: self-referral or referral from family, friends, etc. but no other agency/institution involved; legal: court, probation, police


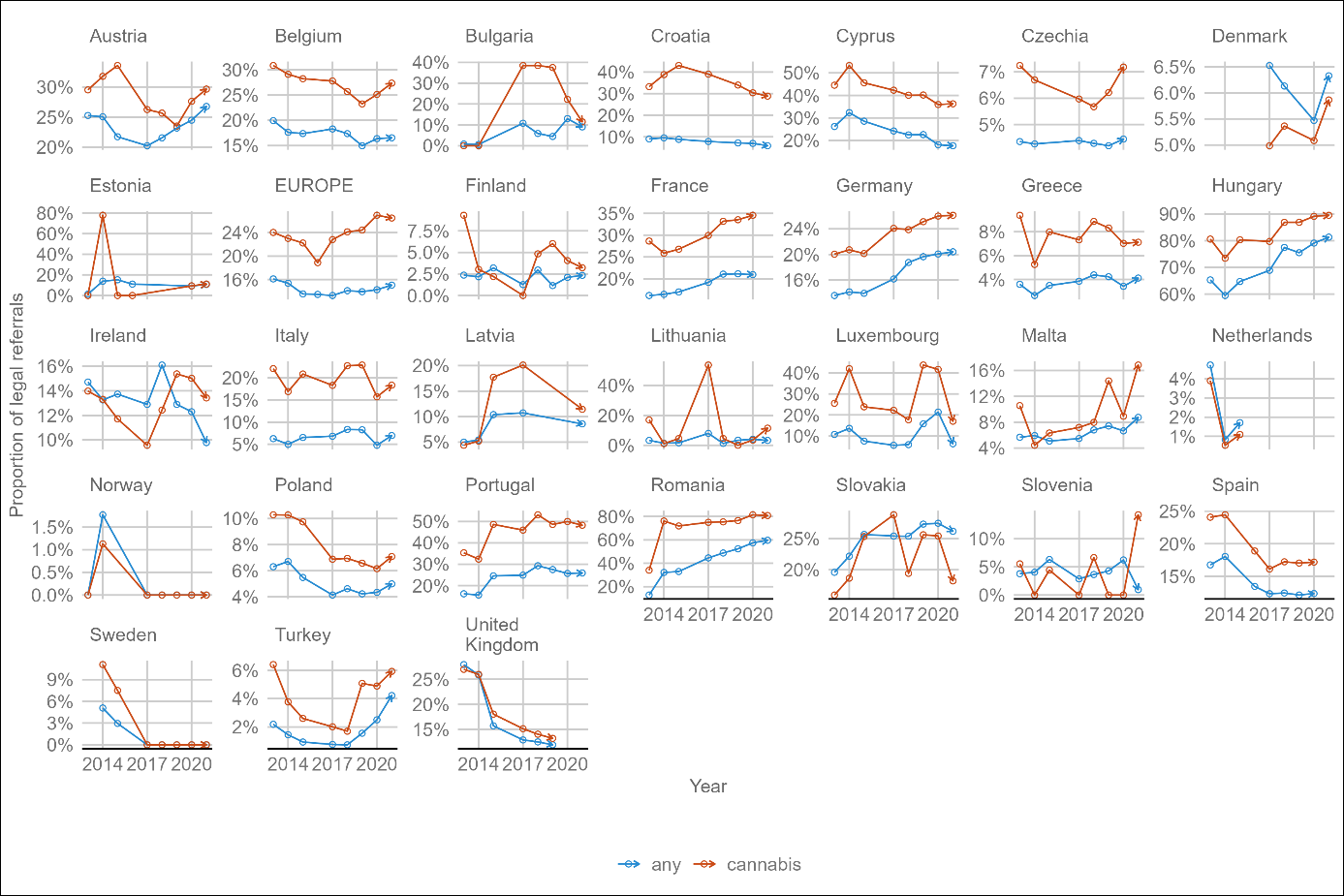


**Supplementary Figure 3**. Proportion of treatment entrants for cannabis (red) or any drug (blue) referred to treatment by legal entities (by court or police or as part of probation) by country for available years since 2013. “EUROPE” refers to the (unweighted) sum of all data available in each year (with contributions of varying countries).
